# Serum Levels of Inflammatory Markers in Depressed Elderly Patients with Diabetes and Mild Cognitive Impairment

**DOI:** 10.1371/journal.pone.0120433

**Published:** 2015-03-20

**Authors:** Malgorzata Gorska-Ciebiada, Malgorzata Saryusz-Wolska, Anna Borkowska, Maciej Ciebiada, Jerzy Loba

**Affiliations:** 1 Department of Internal Medicine and Diabetology, Medical University of Lodz, Lodz, Poland; 2 Department of General and Oncological Pneumology, Medical University of Lodz, Lodz, Poland; University of Naples Federico II, ITALY

## Abstract

**Objective:**

The aim of the study was to determine the serum levels of CRP, IL-6 and TNF-α in elderly diabetic patients with depressive syndrome alone or with coexisting mild cognitive impairment (MCI).

**Methods:**

276 diabetics elders were screened for depressive symptoms (using Geriatric Depression Scale: GDS-30) and MCI (using the Montreal Cognitive Assessment: MoCA score). Data of HbA1c, blood lipids and inflammatory markers levels were collected.

**Results:**

In all groups of patients levels of CRP, IL-6 and TNF-α were significantly higher as compared to controls. The highest level of inflammatory markers was detected in group with depressive mood and coexisting MCI, however IL-6 level didn’t significantly differ as compared to MCI group. We founded correlations between all inflammatory markers in group of patients with depressive mood and in group of subjects with depressive symptoms and coexisting MCI. GDS-30 score was correlated with levels of inflammatory markers in group with depressive mood, and with levels of CRP and TNF-α in group with depressive mood and coexisting MCI. In the group with depressive mood and coexisting MCI we founded that MoCA score was negatively correlated with CRP and TNF-α levels; and HbA1c level was positively correlated with all inflammatory markers. The univariate logistic regression models revealed that variables which increased the likelihood of having been diagnosed with MCI in depressed patients were: higher levels of HbA1c, CRP, IL-6 and TNF-α, previous CVD or stroke, increased number of co-morbidities and microvascular complications, older age, less years of formal education. The multivariable model showed that previous CVD, higher HbA1c and IL-6 levels are significant factors.

**Conclusions:**

We demonstrated that the presence of depressive syndrome is associated with higher levels of inflammatory markers in elderly patients with diabetes. The presence of MCI in these depressed subjects has additive effect on levels of inflammatory mediators.

## Introduction

Diabetes mellitus is a multifactorial metabolic disorder. The underlying etiology, pathophysiology and complications of diabetes are still being elucidated. Recent evidence has indicated that type 2 diabetes mellitus (T2DM) in the elderly is a risk factor for depression, cognitive dysfunction or dementia [1, 2]. A lot of epidemiological and clinical studies have reported the high prevalence of depressive syndrome or mild cognitive impairment (MCI) in patients with diabetes, however there is a very little data about coexistence of these conditions [3, 4]. In our previous study we found the prevalence of MCI was 31.5%; depressive syndrome was 29.7%, and MCI with coexisting depressive mood was 9.1% in elderly diabetic population [5]. There are many hypotheses about the mechanism underlying the linkage between depression and MCI in diabetes. One of the theory tract depression as “vascular disease” and states that cerebrovascular disease can predispose, precipitate or perpetuate a depressive syndrome in older adults [6]. It have been also hypothesized that small vessel diseases in the brain (white matter lesions and lacunae) affect cognitive function and depressive disorders in older diabetics without overt dementia or symptomatic stroke [7]. However other authors had described that association between depressive symptoms and MCI was independent of underlying vascular disease [8]. The researchers had concluded that depressive symptoms in the absence of overt cognitive impairment may reflect the early signs of a neurodegenerative disease or that depression leads to damage in the hippocampus through a glucocorticoid cascade.

One of the hypotheses about depression, cognitive impairment and diabetes linkage is that all these conditions share some common findings: lower hippocampal volumes, vascular changes in the brain and neurotransmitter deficits. All of these changes have been individually linked to chronic inflammation [9]. Depressed patients have been found to have higher levels of proinflammatory cytokines suggesting that inflammatory responses have a crucial role in the pathophysiology of depression [10]. Inflammation may play an important role in the presence and development of MCI [9]. Levels of circulating inflammatory markers are elevated in people with T2DM compared with nondiabetic population. Inflammatory mediators may therefore have a role in the accelerated development of cognitive impairment in people with diabetes either by a direct effect on the brain or through an influence on the development of vascular disease.

C-reactive protein (CRP), tumor necrosis factor- (TNF-α) and interleukin-6 (IL-6) are mediators widely described in worldwide literature have been associated with depression, MCI and diabetes [11, 12, 13]. Two meta-analyses have shown that inflammatory marker levels such as C-reactive protein (CRP), interleukin (IL)-6 and tumor necrosis factor alpha (TNF-α) are increased in depressed persons compared with non-depressed subjects [11, 12].

Depression is accompanied by altered immune function and activation of the inflammatory response system [14]. Activated macrophages secrete pro-inflammatory cytokines, which may contribute to depression. The pro-inflammatory cytokines IL-6 and TNF-*α* have direct inhibitory effects on adult hippocampal neurogenesis [15, 16]. Peripheral/serum levels of IL-6 and TNF-*α* are increased in patients with depression [17, 18], and these effects are normalized following antidepressant treatment [19, 20]. These consistent clinical findings indicate that TNF-*α* and IL-6 are putative biomarkers of depressive episodes and treatment response. There are also evidences from large-study that low-grade chronic inflammatory markers, such as C-reactive protein and interleukin-6 are connected with cognitive symptoms of depression [21].

A longitudinal examination of inflammatory markers and global cognitive decline was conducted in a large sample of older adults from the prospective Health, Aging, and Body Study. Using the Modified Mini-Mental State Examination (3MS) as a measure of cognitive function, the researchers evaluated serum levels of IL-6, CRP, and TNF-α at baseline in relation to baseline cognition and risk of cognitive decline over two years. Findings indicated that participants in the highest tertile of IL-6 or CRP serum concentrations performed significantly worse at baseline and follow-up 3MS, with a 24% increase in risk of cognitive decline over the two year period in comparison to those participants in the lowest tertile [13].

In other population-based study the authors observed significant associations between CRP and MCI and with the attention/executive function domain score [22].

Another large population study analyzed an array of inflammatory blood markers was analyzed including CRP, TNF-α, IL-1 β, IL-6, IL-8, IL-10, IL-12p70, VCAM-1, plasminogen activator inhibitor (PAI-1) and their association with MCI. This study examines also the relationship between systemic inflammation and MCI subtypes. The authors found that higher levels of some inflammatory markers, particularly TNF-α, were found in the MCI group compared to those with normal cognitive function. They also showed a trend that individuals with MCI have higher levels of CRP compared to cognitively normal [23].

Although there are some studies that described separately relationship of inflammation with depressive disorders or with MCI in diabetes, data about common inflammation background in coexisting depressive syndrome and MCI conditions in elderly diabetic population are lacking. Therefore, the aim of the current study was to 1. Determine the serum levels of inflammatory markers (CRP, IL-6 and TNF-α) in elderly diabetic patients with depressive syndrome alone or with coexisting MCI. 2. Examine the associations of these three markers with clinical, depressive and cognitive parameters.

## Materials and Methods

### Study Population

A survey was conducted among unselected 276 elders who attended to outpatient clinic belonged to the Department of Internal Medicine and Diabetology, University Hospital no 1 in Lodz, Poland. A brief screening for recruitment was conducted by the investigators to identify potential participants. We included patients aged 65 and over with diabetes type 2 diagnosed minimum 1 year earlier, subjects who had been able to understand and cooperate with study procedures. The exclusion criteria were: diagnosed depression or dementia, use of possible or known cognition-impairing drugs in the previous 3 month, presence of neoplasm, constant alcohol or substance abuse, severe visual, mobility, or motor coordination impairment, history of head trauma, inflammatory or infectious brain disease, severe neurological or psychiatric illness.

Written consent was obtained from the participants at the beginning of the study. The first part of visit included a morning blood draw after a 10–12 hour overnight fast, blood pressure measurements, height and weight assessment and complete physical examination. Then patients had eaten a breakfast followed by capillary glucose level measuring to ensure that participants were not hypoglycemic at the time of cognitive testing. The second part of visit took place in a private area in the clinic. Subjects completed a questionnaire describing baseline demographics and underwent cognitive testing.

### Participant characteristics, clinical evaluation and risk factor assessment

Demographic variables and possible risk factors were recorded in a standardized interview. Weight and height were measured to calculate body mass index (BMI = weight/ height^2^ [Kg/ m^2^]). The systolic and diastolic blood pressures (mmHg) were measured with the patient in sitting position after 5 minutes of rest. The detailed medical history of diabetes type 2 was taken and includes: diabetes duration, currant treatment for diabetes and complications if present, family history of diabetes, co-morbid diseases of the patient (hyperlipidemia, hypertension, cardiovascular disease, lung disease, cancer, gastrointestinal tract diseases) and their treatment. Educational level was recorded in years of education. Smoking was classified as current, past, or never. Physical activity was recorded if any present. Diabetic vascular complications were assessed based on the existence of nephropathy, retinopathy, neuropathy, cardiovascular disease (CVD) and stroke. Hypertension was defined as either a history of hypertension or use of any antihypertensive agents, Hyperlipidemia defined as use of any lipid lowering agent or an untreated serum LDL cholesterol level 2.6 mmol/l or/and triglycerides 1.7 mmol/l.

### Blood biochemistry

After overnight fasting, blood samples were taken by venipuncture to assess serum levels of glycosylated hemoglobin (HbA1c), total cholesterol, triglycerides, low-density lipoprotein cholesterol (LDL-C) and high-density lipoprotein cholesterol (HDL-C). All the parameters were measured in a centralized laboratory.

### Determination of serum CRP, IL-6 and TNF-α

The serum levels of CRP, IL-6 and TNF-α were determined by Quantikine Human Immunoassay ELISA kit (R & D System, Minneapolis, USA) according to the instructions of the manufacturer. Minimum detectable concentrations were: 1x10^−5^ mg/L for CRP, 0.7 pg/ml for IL-6 and 1.6 pg/ml for TNF-α.

### Neuropsychological evaluations

All participants underwent the following tests: the Montreal Cognitive Assessment (MoCA) [24] to evaluate the cognitive impairment, long version of the Geriatric Depression Scale (GDS-30) [25] to assessed the depressive mood, Katz Basic Activities of Daily living (BADL) and Lawton Instrumental Activities of Daily Living (IADL) questionnaires to collect information on daily activities [26, 27]. The MoCA tests 8 cognitive domains, visual-spatial ability, attention, executive function, immediate memory, delayed memory, language, abstraction, calculation, and orientation, for a maximum total score of 30. The normal MoCA score is ≥26, with one point added if the subject has fewer than 12 years of formal education. The MoCA is better than other tools to detect MCI in the elderly patients with type 2 diabetes [28]. MCI was diagnosed based on criteria established the 2006 European Alzheimer’s

Disease Consortium which are currently available standard test [29, 30]. These criteria included absence of dementia. The cut-off points for MoCA scores (19/30) are recommended for the diagnosis of ‘dementia’ in epidemiological studies. Patients with score 19 and below were excluded from the study as dementia and sent to psychiatrist for further care. The criteria mentioned above included also absence of major repercussions on daily life (in our study, measured by Katz BADL and Lawton IADL).

This interview was followed by Geriatric Depression Scale (GDS) for mood assessment [25]. GDS consists of 30 items. Scores range from 0 to 9 was considered as normal, 10 to 19 was considered to have depressive symptoms. Score 20 and above was excluded from the study as severe depressive symptomatology and sent to psychiatrist for further diagnosis.

According to criteria mentioned above 276 older subjects with diabetes type 2 were selected into groups: patients with depressive mood, patients with MCI and depressive mood, patients with MCI, controls (patients without MCI and without depressive mood).

### Ethics

The study was operated in accordance with the World Medical Association’s Declaration of Helsinki. Each participant was assigned a number by which he/she was identified to keep his or her privacy. The approval was obtained from the independent local ethics committee of Medical University of Lodz No RNN/420/13/KB. The purpose, nature, and potential risks of the experiments were fully explained to the subjects, and all subjects gave written, informed consent at the beginning of the study. The subjects had the full capacity to consent because they maintained general cognitive function and daily activities. We included only patients who had been fully able to understand and cooperate with study procedures. We excluded subjects with diagnosed depression or dementia. There wasn’t any surrogate consent procedure (e.g., whereby next of kin or legally authorized representative) consented on the behalf of participants.

## Statistical Analysis

All data are presented as means ± SD. Normality of distributions was assessed using the Shapiro-Wilk tests. The descriptive statistics the continuous variables using the Student’s t or the Mann Whitney-U tests whenever applicable. From parametric methods comparison between groups was done with one-way ANOVA test followed by post-hoc test. From nonparametric methods comparison between groups was done with Kruskal-Wallis ANOVA followed by post-hoc test. Pearson correlation analysis for normally distributed variables and Spearman rank correlation for nonnormally distributed variables were used to assess relationships. Simple logistic regression model was done in order to select so-called independent factors which increase the selection risk of MCI in patients with depressive symptoms. Then multivariable regression model in order to select the “strongest” factor from independent risk factors. To “optimize” the multivariable model, a stepwise approach was used (backward elimination with Wald criteria). Odds ratios (OR) were computed and presented with the 95% interval of confidence (CI). A P value of less than 0.05 was considered statistically significant. Statistica 10.0 (StatSoft, Poland, Krakow) was used for analysis.

## Results

### Clinical characteristics

The demographic and clinical characteristics of the study group have been presented in Table 1. According to criteria mentioned above we selected 57 patients with depressive mood, 25 patients with MCI and depressive mood, 62 patients with MCI and 132 controls. Compared with controls patients with depressive mood were significantly female, single, had more often current and past smoking habits, had less physical activity, higher BMI, a longer duration of diabetes, more were diagnosed with neuropathy, hiperlipidemia and other co-morbidities, had a history of hypoglycemia and were treated with insulin. Compared with controls patients with depressive mood and MCI were older, female, single, less educated, had more often past smoking status, had less physical activity, higher BMI, a longer duration of diabetes, more were diagnosed with neuropathy, retinopathy, hiperlipidemia and other co-morbidities, had a history of hypoglycemia and were treated with insulin. The characterization of 62 subjects with MCI is presented in Table 1.

**Table 1:** Demographic and clinical characteristics of type 2 diabetic elderly patients.

	All subjects	depressive syndrome	depressive syndrome and MCI	MCI	Controls	Difference (p<0.001)
Number of patients	276	57	25	62	132	
Male/female	127/149	11/46	4/21	30/32	50/82	b, c, d, e
Age (years)	73.6±4.8	72.9±4.0	78.0 ± 5.3	74.7±3.9	72.5±4.9	a, d, e, f
Education-years	11.3 ±2.4	12.1±2.3	9.5 ± 1.7	9.8±1.9	12.1±2.2	a, b, e, f
Marital status: single/married	127/149	37/20	21/4	24/38	45/87	b, c, d, e
Current smoking	19 (6.88%)	9 (15.7%)	4 (16%)	2 (3.2%)	4 (3.0%)	b, c
Had ever smoked	93 (33.7%)	33 (57.8%)	14 (56%)	12 (19.4%)	34 (25.7%)	b, c, d, e
Lack of physical activity (%)	105 (38.0%)	43 (75.43%)	14 (56%)	12 (19.4%)	36 (27.3%)	b, c, d, e
BMI (kg/m^2^)	29.9±3.67	32.1±3.7	31.8 ± 3.2	29.8±3.5	28.6±3.1	b, c, e
Duration of DM2 (years)	8.69 ± 6.23	9.96±6.8	12.8 ± 6.23	10.63±6.2	6.45±5.07	c, e, f
Treatment Insulin	130(47.1%)	41 (71.9%)	19 (76%)	23 (37%)	47 (35.6%)	b, c, d, e
OAD	222(80.4%)	46 ((80.7%)	20 (80%)	61 (98%)	126 (95.4%)	b, c, d, e
Previous CVD	109(39.5%)	10 (17.54%)	23 (92%)	48 (77.4%)	28 (21.2%)	a, b, e, f
Stroke	14 (5.07%)	2 ((3.5%)	5 (20%)	2 (3.2%)	5 (3.78%)	a, d, e
Previous HA/ use of HA drugs	213(77.17%)	39 (68.42%)	21 (84%)	60 (96.7%)	93 (70.4%)	b, f
Hiperlipidemia	218 (78.9%)	52 (91.22%)	24 (96%)	56 (90.3%)	86 (65.15%)	c, e, f
Retinopathy	121(43.8%)	17 (29.8%)	18 (72%)	43 (69.4%)	43 (32.6%)	a, e
Nephropathy	97 (35.1%)	16 (28.07%)	11 (44%)	32 (51.6%)	38 (28.8%)	b, f
Neuropathy	56 (20.2%)	23 (40.35%)	10 (40%)	10 (16.1%)	13 (9.8%)	b, c, e
Co-morbidity (n)	4.66 ± 3.11	5.3±2.5	9 ± 2.82	6.3±3.06	2.8±1.8	a, c, d, e, f
Hypoglycemia	117 (42.3%)	38 (66.6%)	22 (88%)	38 (61.3%)	19 (14.4%)	c, e, f
MoCA score	25.6±3.07	27.86±1.6	21.8±1.7	21.5±1.5	27.3±1.2	a, b, e, f
GDS-30 score	6.8±6.5	15.9±2.8	16±2.7	3.6±2.7	2.7±2.6	b, c, d, e
Katz BADL score	4.96±0.2	4.965±0.1	4.96±0.2	5±0.2	4.96±0.2	
Lawton IADL score	7.9±0.1	7.8±0.1	7.96±0.02	8±0.1	7.9±0.08	
Other diseases: Lung disease (%)	37 (13.4%)	8 (14.04%)	4 (16%)	11 (17.7%)	14 (10.6%)	
Atrial fibrillation (%)	57 (20.6%)	14 (24.6%)	7 (28%)	14 (22.6%)	22 (16.6%)	
Heart failure (%)	58 (21%)	13 (22.8%)	7 (28%)	16 (25.8%)	22 (16.7%)	
Gastrointerstinal tract disease (%)	110 (39.8%)	27 (47.3%)	13 (52%)	29 (46.7%)	41 (31.1%)	
Kidney disease (%)	60 (21.7%)	14 (24.5%)	7 (28%)	15 (24.2%)	60 (21.7%)	
Thyroid diasease (%)	74 (26.8%)	18 (31.6%)	18 (31.6%)	17 (27.4%)	30 (22.7%)	
Other treatment: Angiotensin-converting enzyme inhibitors (%)	130 (47.1%)	27 (47.4%)	12 (48%)	35 (56.4%)	56 (42.4%)	
Angiotensin II receptor blockers (%)	100 (36.2%)	17 (29.8%)	9 (36%)	25 (40.3%)	49 (37.1%)	
Diuretics (%)	87 (31.5%)	21 (36.8%)	11 (44%)	23 (37.1%)	32 (24.2%)	
Calcium channel blockers (%)	87 (31.5%)	19 (33.3%)	9 (36%)	20 (32.3%)	39 (29.5%)	
a1-Blockers (%)	29 (10.5%)	7 (12.2%)	4 (16%)	7 (11.2%)	11 (8.3%)	
B-blockers (%)	132 (47.8%)	21 (36.8%)	20 (80%)	43 (69.4%)	48 (36.3%)	a, b, e, f,
Antiplatelet medications (%)	175 (63.6%)	34 (59.6%)	24 (96%)	48 (77.4%)	69 (52.2%)	a, e, f
Lipid-lowering medications (%)	191 (69.2%)	40 (70.1%)	20 (80%)	42 (67.7%)	89 (67.4%)	

a- comparing: depressive syndrome—MCI and depressive syndrome

b- comparing: depressive syndrome—MCI

c- comparing: depressive syndrome—controls

d- comparing: MCI and depressive syndrome—MCI

e- comparing: MCI and depressive syndrome-controls

f- comparing: MCI—controls

DM2—diabetes type 2, OAD- oral anti-diabetic drug, CVD—cardiovascular disease, HA- hypertension, BMI—body mass index, MoCA—Montreal Cognitive Assessment, GDS—Geriatric Depression Scale, BADL—Basic Activities of Daily living, IADL—Instrumental Activities of Daily Living

The ANOVA test followed by post-hoc test was used to test for significant differences

### The neuropsychological scores

GDS-30 score was significantly higher in patients with depressive mood and in group of subjects with depressive mood and coexisting MCI as compared to controls and patients with MCI.

MoCA score was the lowest in group with MCI and in group with depressive mood and MCI. Patients with depressive syndrome and controls had normal MoCA values. Data are presented in Table 1.

### Biochemical parameters

The mean HbA1c level didn’t differ between patients with depressive syndrome and controls. The level of HbA1c was significantly higher in patients with depressive mood and MCI as well as in MCI group. The level of total cholesterol and LDL cholesterol was the highest in group with depressive mood and higher in group with depressive mood and MCI as compared to controls. The level of triglycerides was highest and HDL was the lowest in group with depressive mood and coexisting MCI. Data are presented in Table 2.

**Table 2:** Inflammatory markers and biochemical characteristics of type 2 diabetic elderly patients.

	All subjects	depressive syndrome	depressive syndrome and MCI	MCI	Controls	Difference (p<0.001)
Nr of patients	276	57	25	62	132	
CRP (mg/L)	5.08±2.8	5.52±2.5	9.77±3.3	6.72±1.7	3.24±1.3	a, b, c, d, e, f
IL-6 (pg/ml)	4.68±2.5	5.22±1.7	8.39±1.9	6.87±1.2	2.71±1.2	a, b, c, e, f
TNF-α (pg/ml)	5.33±2.6	5.51±2.1	9.02±2.4	7.3±1.8	3.63±1.5	a, b, c, d, e, f
HbA1c (%)	7.24±0.68	7.06±0.6	8.02 ± 0.68	7.62±0.69	7±0.5	a, b, e, f
CHOL (mmol/L)	10.3±2.18	11.96±2.12	10.75± 2.1	10.13±2.2	9.58±1.76	b, c, e
LDL (mmol/L)	6.06±1.67	7.1±1.8	6.54 ± 1.89	5.79±1.49	5.64±1.5	b, c, e
TG (mmol/L)	9.65±2.23	9.16±1.98	11.33±2.63	10.29±2.66	9.24±1.79	a, b, e, f
HDL (mmol/L)	2.5± 0.51	2.69±0.37	2.15 ± 0.45	2.36±0.64	2.66±0.44	a, b, e, f

a- comparing: depressive syndrome—MCI and depressive syndrome;

b- comparing: depressive syndrome—MCI

c- comparing: depressive syndrome—controls

d- comparing: MCI and depressive syndrome—MCI

e- comparing: MCI and depressive syndrome-controls

f- comparing: MCI—controls

DM2—diabetes type 2, OAD- oral anti-diabetic drug, CVD—cardiovascular disease, HA- hypertension, BMI—body mass index, MoCA—Montreal Cognitive Assessment, GDS—Geriatric Depression Scale

The ANOVA test followed by post-hoc test was used to test for significant differences

### Inflammatory markers

**Fig 1:**
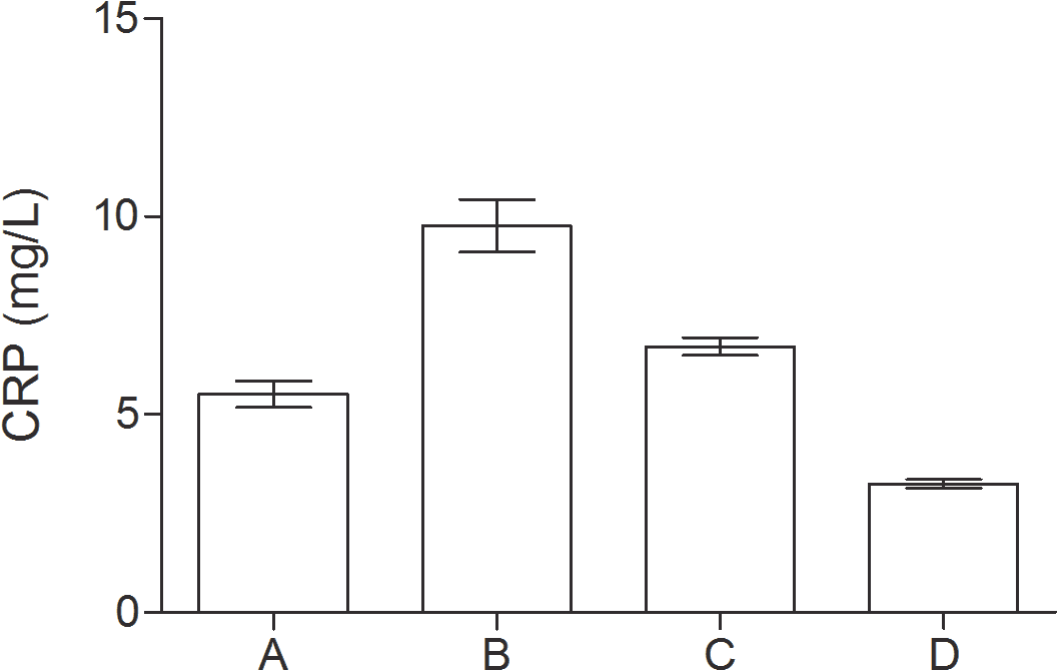
Serum levels of CRP (mg/L) in group of diabetic elderly patients. A—depressive syndrome; B—depressive syndrome and coexisting MCI; C—MCI; D—controls.

**Fig 2:**
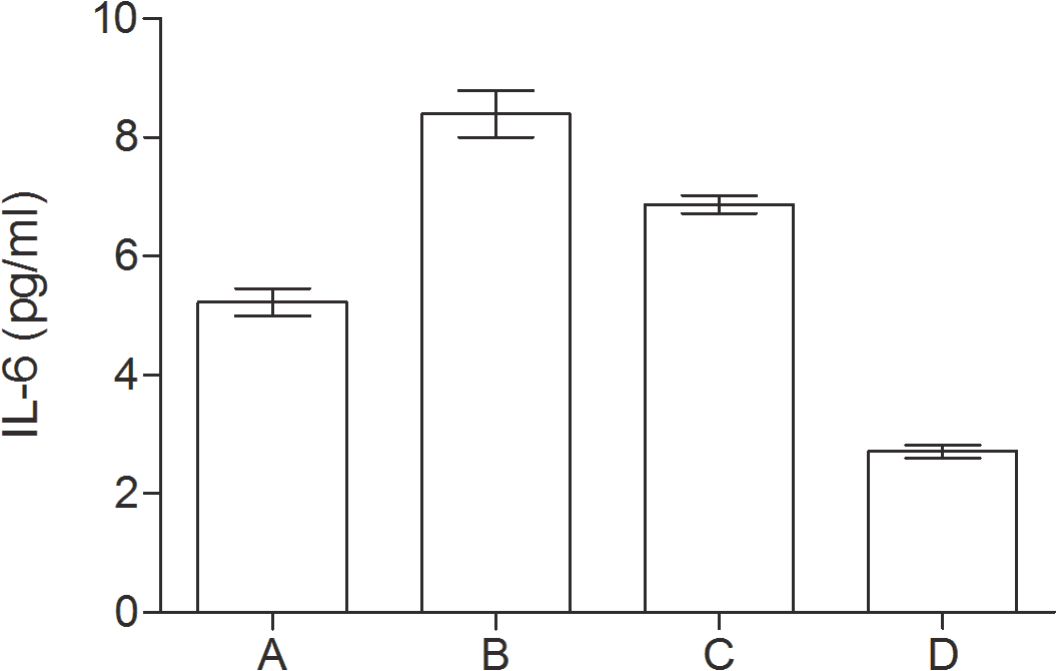
Serum levels of IL-6 (pg/ml) in group of diabetic elderly patients. A—depressive syndrome; B—depressive syndrome and coexisting MCI; C—MCI; D—controls.

**Fig 3:**
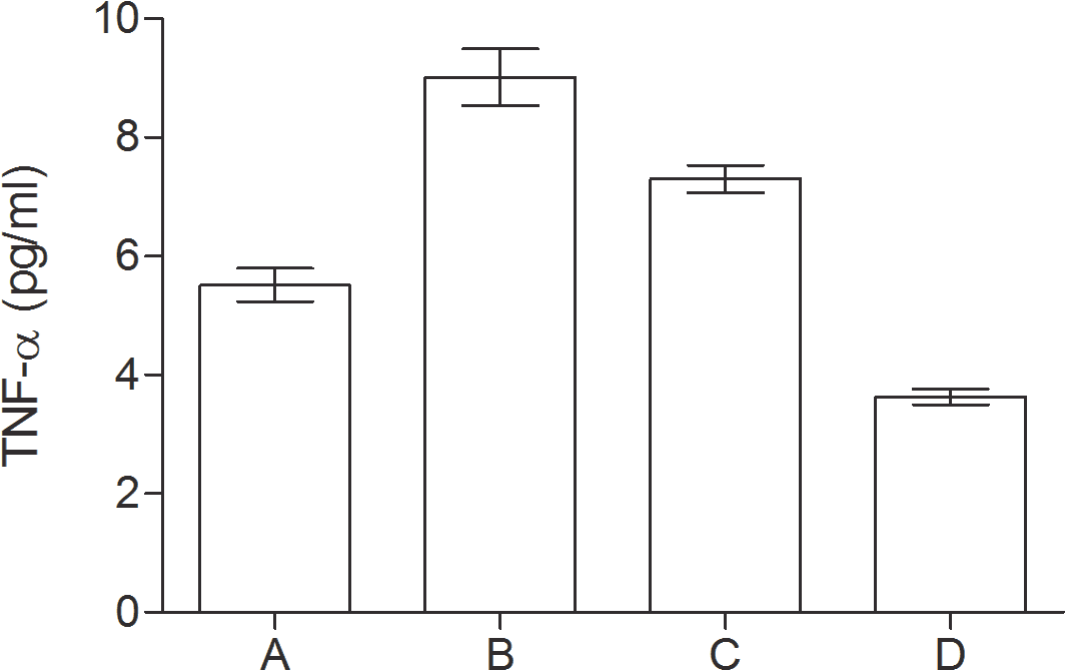
Serum levels of TNF-α (pg/ml) in group of diabetic elderly patients. A—depressive syndrome; B—depressive syndrome and coexisting MCI; C—MCI; D—controls.

In all groups of patients with psychological disturbances the serum levels of CRP, IL-6 and TNF-α were significantly higher as compared to controls. The highest level of inflammatory markers was detected in group with depressive mood and coexisting MCI as compared to other groups, however IL-6 level didn’t significantly differ as compared to MCI group. Data are presented in Table 2 and in Figs. 1, 2 and 3.

Because the onset time of the diabetes evaluation could be fundamental to assess the burden of inflammation in studies groups we dived each group into 2: elderly onset—diabetes diagnosis at the age 65 and older; and middle age onset—diabetes diagnosis below 65 years old). We didn’t find any significant differences between elderly and middle age onset in each group (using the Student’s t or the Mann Whitney-U tests). Then we compared all parameters between all four groups (patients with depressive mood, patients with MCI and depressive mood, patients with MCI, controls) first—in elderly onset of diagnosis of diabetes, second—in middle age onset of diagnosis of diabetes (using ANOVA test followed by post-hoc test). We founded all differences similar to the statistics describe above without division for elderly and middle age onset of diagnosis of diabetes. All data are presented in Table 3.

**Table 3:** Inflammatory markers and biochemical characteristics of type 2 diabetic elderly patients in dependence on elderly or middle age onset of the diagnosis of the diabetes.

	All subjects	All subjects	depressive syndrome	depressive syndrome	depressive syndrome and MCI	depressive syndrome and MCI	MCI	MCI	Controls	Controls	Difference (p<0.001)
Onset of the diagnosis of the diabetes	middle age	elderly age	middle age	elderly age	middle age	elderly age	middle age	elderly age	middle age	elderly age	
Nr of patients	115	161	31	26	11	14	28	34	45	87	
CRP (mg/L)	5.4±2.6	4.9±2.9	5.9±2.3	4.9±2.6	9±3.1	10.3±3.4	6.7±1.7	6.7±1.8	3.4±1.3	3.2±1.3	a, b, c, d, e, f
IL-6 (pg/ml)	4.8±2.3	4.5±2.6	5.3±1.7	5.1±1.6	7.6±1.4	8.9±2.1	6.9±1.1	6.8±1.2	2.7±1.2	2.7±1.3	a, b, c, e, f
TNF-α (pg/ml)	5.6±2.5	5.1±2.6	5.8±2.1	5.1±2.0	8.7±2.1	9.2±2.6	7.3±1.7	7.2±1.8	3.6±1.5	3.6±1.6	a, b, c, d, e, f
HbA1c (%)	7.3±0.6	7.1±0.6	7.1±0.6	6.9±0.6	7.9±0.7	8.1±0.7	7.7±0.7	7.6±0.6	7.1±0.5	6.9±0.4	a, b, e, f
CHOL (mmol/L)	10.4±2.2	10.1±2.1	12.0±2.1	11.8±2.2	11.2±2.2	10.3±2.1	9.7±2	10.4±2.3	9.7±1.9	9.5±1.6	b, c, e
LDL (mmol/L)	6.2±1.6	5.8±1.6	7.2±1.7	6.9±1.8	7.1±2.1	6.1±1.7	5.5±1	6±1.7	5.9±1.5	5.4±1.4	b, c, e
TG (mmol/L)	9.5±2.5	9.7±1.9	9.1±2.3	9.2±1.6	11.4±2.8	11.2±2.5	9.4±3	10.9±2.1	9.3±2.1	9.2±1.5	a, b, e, f
HDL (mmol/L)	2.6±0.5	2.4±0.4	2.7±0.3	2.6±0.4	2.2±0.5	2.1±0.4	2.5±0.7	2.1±0.5	2.7±0.4	2.6±0.4	a, b, e, f

a- comparing: depressive syndrome—MCI and depressive syndrome

b- comparing: depressive syndrome—MCI

c- comparing: depressive syndrome—controls

d- comparing: MCI and depressive syndrome—MCI

e- comparing: MCI and depressive syndrome-controls

f- comparing: MCI—controls

DM2—diabetes type 2, OAD- oral anti-diabetic drug, CVD—cardiovascular disease, HA- hypertension, BMI—body mass index, MoCA—Montreal Cognitive Assessment, GDS—Geriatric Depression Scale the Student’s or the Mann Whitney-U tests were used to assess differences between elderly and middle age onset in each group; The ANOVA test followed by post-hoc test was used to test for significant differences between all four groups: first—in elderly onset of diagnosis of diabetes, second—in middle age onset of diagnosis of diabetes.

### Correlations

We founded significant correlations between CRP and IL-6, CRP and TNF-α, IL-6 and TNF-α in group of patient with depressive mood as well as in group of subject with depressive symptoms and coexisting MCI. The results indicated that GDS-30 score was positively correlated with serum levels of CRP, IL-6 and TNF-α in group with depressive mood, and with serum levels of CRP and TNF-α in group with depressive mood and coexisting MCI. In the group with depressive mood and coexisting MCI we founded that MoCA score was negatively correlated with CRP and TNF-α levels; and HbA1c level was positively correlated with all inflammatory markers. All correlations are presented in Table 4 and Table 5. To better understand correlation we constructed logistic regression models (see below).

**Table 4:** Correlations coefficients among serum levels of CRP, IL-6, TNF-α, HbA1c, lipids, BMI and GDS-30 score in group of diabetic elderly patients with depressive mood.

	IL-6 (pg/ml)	TNF-α (pg/ml)	GDS-30 score	HbA1c (%)	CHOL (mmol/L)	LDL (mmol/L)	HDL (mmol/L)	TG (mmol/L)	BMI (kg/m^2^)
CRP	0.51***	0.64***	0.58***	0.18	0.46***	0.08	0.26*	0.05	0.49***
IL-6	1	0.51***	0.39**	0.095	0.34*	0.03	-0.005	0.12	0.25*
TNF-α		1	0.57***	0.19	0.55***	0.27*	-0.05	0.1	0.51***
GDS-30 score			1	0.024	0.31*	0.29*	-0.16	0.03	0.46***

*P<0.05

** p<0.01

***p<0.001; BMI—body mass index, GDS—Geriatric Depression Scale

**Table 5:** Correlations coefficients among serum levels of CRP, IL-6, TNF-α, HbA1c, lipids, BMI, MoCA and GDS-30 score in group of diabetic elderly patients with depressive mood and coexisting MCI.

	IL-6 (pg/ml)	TNF-α (pg/ml)	GDS-30 score	MoCA score	HbA1c (%)	CHOL (mmol/L)	LDL (mmol/L)	HDL (mmol/L)	TG (mmol/L)	BMI (kg/m^2^)
CRP	0.52***	0.58**	0.56***	-0.56**	0.74***	0.49*	0.04	-0.28	0.15	0.31
IL-6	1	0.56**	0.35	-0.25	0.58**	0.03	0.11	-0.52**	0.33	0.1
TNF-α		1	0.77***	-0.45*	0.65***	0.32	0.38	-0.65***	0.36	0.55**
GDS-score			1	-0.63**	0.57**	0.38	0.25	-0.23	-0.01	0.43*
MoCA score				1	-0.55**	-0.18	0.1	0.14	0.09	-0.33

*P<0.05

** p<0.01

***p<0.001; BMI—body mass index, MoCA—Montreal Cognitive Assessment, GDS—Geriatric Depression Scale

### Logistic regression models

Because many factors (like vascular co-morbidities, cardiovascular risk factors, duration of DM, age etc.) can influence the results we constructed the univariate logistic regression models and finally multivariable regression model to determine the predictors of having MCI in depressed diabetic patients. The independent variables entered in the model at step one were: demographic variables (age, gender, education), duration of diabetes, glycaemic control (HbA1c level), cardiovascular diseases (MI, angina, stroke), cardiovascular risk factors (BMI, smoking status, hiperlipidemia, previous HA or use of HA drugs), microvascular complications, number of co-morbid conditions, levels of CRP, IL-6 and TNF-α. The univariate logistic regression models revealed that variables which increased the likelihood of having been diagnosed with MCI in depressed patients with diabetes were: higher levels of HbA1c, CRP, IL-6 and TNF-α, previous CVD or stroke, increased number of co-morbidities and microvascular complications, older age and less years of formal education (Table 6).

**Table 6:** Assessment results of the risk of having MCI in a simple logistic regression model in the patients with depressive syndrome.

Variables analyzed	ß	SE of ß	p value	OR	95% CI
Age (years)*	0.24	0.06	P<0.001	1.27	1.12–1.44
Gender: female	-0.14	0.03	0.72	0.89	0.47–1.67
Education (years)*	-0.69	0.16	P<0.001	0.5	0.36–0.69
Current smoking	0.02	0.001	P = 0.98	1.01	0.52–1.91
Duration of DM2 (years)	0.06	0.03	P = 0.09	1.06	0.99–1.14
Previous stroke*	0.96	0.43	P = 0.028	2.62	1.11–6.19
Previous CVD*	1.46	0.31	P<0.001	4.34	2.38–7.87
Previous HA or use of HA drugs	0.44	0.31	P = 0.15	1.56	0.85–2.84
Hiperlipidaaemia	0.42	0.56	P = 0.45	1.52	0.51–4.56
Microvascular complications (n)*	0.83	0.31	P = 0.008	2.29	1.24–4.22
Co-morbidity (n)*	0.55	0.13	P<0.001	1.75	1.34–2.28
BMI (kg/m2)	-0.02	0.06	0.79	0.98	0.86–1.12
HbA1c (%)*	1.93	0.43	P<0.001	6.93	2.95–16.24
CRP (mg/L)*	0.68	0.16	P<0.001	1.98	1.42–2.75
IL-6 (pg/ml)*	1.27	0.32	P<0.001	3.58	1.91–6.69
TNF-α (pg/ml)*	0.78	0.19	P<0.001	2.18	1.48–3.22

Abbreviations: ß: regression coefficient; CI: confidence interval for odds ratio; OR: odds ratio; SE: standard error

*significance, p<0.05

Table 7 shows the results of modeling the risk of having MCI by multivariable regression. All variables presented in Table 7 were introduced to this model. The multivariable model was optimized by the stepwise approach. Previous CVD, poorer glycaemic control (higher HbA1c level) and higher level of IL-6 are the factors increasing the likelihood of having MCI in depressed patients. However the previous CVD was the strongest factor.

**Table 7:** Assessment results of the risk of having MCI in a multivariable logistic regression model in the patients with depressive syndrome.

Variables analyzed	ß	SE of ß	p value	OR	95% CI
Previous CVD*	1.38	0.44	P = 0.002	3.99	1.67–9.54
HbA1c (%)*	1.31	0.59	P = 0.027	3.69	1.16–11.7
IL-6 (pg/ml) *	1.01	0.4	P = 0.011	2.76	1.25–6.06

Abbreviations: ß: regression coefficient; CI: confidence interval for odds ratio; OR: odds ratio; SE: standard error

*significance, p<0.05

## Discussion

Clinical studies have indicated a frequent coexistence of depression and diabetes. Depressive disorders are associated with increased medical morbidity and mortality in individuals with diabetes. It was demonstrated that there is a higher prevalence of diabetes complications, including retinopathy, nephropathy, neuropathy and macrovascular complications among individuals with diabetes and depression compared to those without depression [31]. Depression is a disease with a poorly recognized etiology. Participation of the immune system, especially excessive activation of the pro-inflammatory cytokines in the central nervous system in the pathogenesis of depression, has also been well documented [32].

We found that serum levels of CRP, IL-6 and TNF-α in diabetic elderly patients with depressive symptoms were significantly higher as compared to controls. More over our results indicated that levels of these inflammatory markers were positively correlated with GDS-30 score. Our results are consistent with many other studies have found associations between depression and several cytokines and inflammatory mediators. One large cross-sectional study compared four groups of subjects: patients with T2DM and elevated depressive symptoms; patients with T2DM, patients with depressive symptoms and healthy controls. The authors showed that those with T2DM and elevated depressive symptoms have significantly higher levels of IL-6 compared to all others groups [33]. Findings from this study also demonstrate that the presence of T2DM, but not elevated depressive symptoms, is associated with higher levels of CRP. However the authors didn’t observe the presence of an additive effect of T2DM and elevated depressive symptoms on TNF-α. Other studies showed that clinical depression and depressive symptoms are positively correlated with CRP levels after controlling for age, gender, BMI, high density lipoprotein cholesterol concentrations, and other traditional risk factors of depression [34, 35]. C-reactive protein—an acute-phase protein is the most commonly used inflammatory biomarker. Its expression is mainly regulated by IL-6 during the acute phase with both a causative role of DM and a role associated with the risk of DM complications. Other inflammatory cytokines participating in low grade inflammation in diabetes are IL-6 and TNF- α, with a role that is mainly associated with the risk of DM complications. The increase in pro-inflammatory mediators detailed above provides a plausible mechanistic link explaining the increased incidence of depression among patients with T2DM. The authors had proposed four possible pathways that cytokines may use to gain access to the central nervous and exert an influence on behavior [32]. In one pathway, cytokines interact with specific receptors on afferent peripheral nerves including the vagus nerve resulting in neuronal transduction of the signal into the central nervous system where they activate microglia to produce cytokines centrally [36]. Another pathway involves saturable cytokine transporters that allow for a direct transfer of many cytokines across the blood–brain barrier. Once the cytokine signal has crossed the blood–brain barrier interacts with several brain mechanisms to induce depressive symptoms [37].

It has been hypothesized that explain the association between depressive syndrome and cognitive impairment occur in patients with diabetes results from the chronic inflammatory changes that are linked to the activation of macrophages in the blood and microglia in the brain [38]. Our study demonstrated that the highest level of inflammatory markers was detected in group with depressive mood and coexisting MCI as compared to other groups. Moreover we found correlations between some inflammatory mediators and GDS-30 and MoCA scores. These observations are a novelty in literature. They suggest that the presence of MCI in these depressed subjects have additive effect on inflammation.

However there are many studies which investigate inflammatory mediators in MCI and diabetes. In one study the authors demonstrated that plasma CRP is associated with prevalent MCI in elderly, nondemented subjects. These findings suggest an involvement of inflammation in the pathogenesis of MCI given the role of CRP as a marker of inflammation and of vascular risk [22]. Other large population study showed that elevated circulating levels of inflammatory markers (CRP, IL-6 and TNF- α) were associated with poorer cognitive ability in elderly patients with T2DM [39]. Our results indicate that low—grade inflammation could be a possible link between these psychiatric disorders in diabetes. Several studies have reported higher serum levels of the inflammatory cytokines IL-6, IL-1 and TNF- α in depression and AD [40]. IL-6 is a cytokine that mediates immune responses and inflammatory reactions, affecting CNS cell growth and differentiation. IL-6 overexpression is generally detrimental and destructive to cell growth. CRP has been found in and around amyloid plaques and around small-vessel damages in MCI patients [9].

In our study the univariate logistic regression models revealed that factors variables which increased the likelihood of having been diagnosed with MCI in depressed patients with diabetes were: higher levels of HbA1c, CRP, IL-6 and TNF-α, previous CVD or stroke, increased number of co-morbidities and microvascular complications, older age and less years of formal education. In the multivariable analysis we found the factors increasing the likelihood of having MCI in depressed patients: previous CVD (the strongest factor), poorer glycaemic control (higher HbA1c level) and higher level of IL-6. The results suggest that the higher levels of inflammatory markers in depresses patients are accounted for by vascular co-morbidities. Epidemiological studies have reported an association between depressive symptoms and subclinical atherosclerosis, including carotid IMT and carotid plaques [41, 42, 43, 44]. Although in our study is we didn’t assess the carotid intima-media thickness, we included all factors which can contributes to higher inflammation (etc. cardiovascular diseases, microvascular complications, number of co-morbid conditions) into logistic analysis. Vascular pathology is has been observed in patients with depressive symptoms in large epidemiologic studies [45], as well as in MCI patients [46]. Inflammation and immune dysregulation have been critically involved in vascular disease in pathogenesis of diabetes complications [47]. Prior research has suggested that markers of inflammation may be a cause or consequence of elevated depressive symptoms found among patients with other types of inflammatory-related diseases (e.g., cardiovascular disease) [48, 49, 50, 51].

Inflammation can follow, precipitate or aggravate vascular events. Therefore, inflammation has a bidirectional role in the link between depression, MCI and vascular pathology [9]. Thus, prospective design studies are needed to better understand the temporal associations between inflammation and these co-morbidities.

The higher inflammatory status could be related to a metabolic syndrome. However our analysis didn’t revealed any influence of single components of metabolic syndrome (BMI, smoking status, hiperlipidemia, previous HA or use of HA drugs) we showed that CVD history is the strongest factor related to diagnosis of MCI in depressed diabetics. The metabolic syndrome is a predictor of cardiovascular events including coronary heart disease and stroke [52]. Our results are consistent to other studies. The authors found no overall association between metabolic syndrome and MCI, but they have previously observed, that vascular phenomena such as diabetes, stroke, and coronary heart disease are associated with non amnestic MCI [53]. They concluded in the presence of inflammation, the metabolic syndrome may be more likely to be associated with the form of cognitive impairment—nonamnestic-MCI, that may be more closely linked to cerebrovascular disease.

## Conclusions

Findings from this study demonstrate that the presence of depressive syndrome is associated with higher levels of inflammatory markers in elderly patients with diabetes. The presence of MCI in these depressed subjects has additive effect on levels of inflammatory mediators. Previous CVD, poorer glycaemic control (higher HbA1c level) and highest level of IL-6 are the factors increasing the likelihood of having MCI in depressed patients.

However there are data that suggest that inflammatory mediators are involved in the development of depressive symptoms and cognitive impairment in diabetes the causative mechanisms of diabetes on brain’s complication seems to be very complicated. Since inflammation could be a cause or consequence of T2DM, elevated depressive symptoms and cognitive impairment more prospective design research is needed to understand the temporal associations between inflammation and these co-morbidities. Better understanding of the role of inflammation in depression and cognitive impairment could results in the rational development of immunomodulatory strategies and the improvement of the effectiveness of the treatment.
